# Achieving minimal disease activity in psoriatic arthritis predicts meaningful improvements in patients’ health-related quality of life and productivity

**DOI:** 10.1186/s41927-018-0030-y

**Published:** 2018-08-13

**Authors:** Laura C. Coates, Ana-Maria Orbai, Akimichi Morita, Olivier Benichou, Lisa Kerr, David H. Adams, Catherine L. Shuler, Julie Birt, Philip S. Helliwell

**Affiliations:** 1 Nuffield Department of Orthopaedics, Rheumatology and Musculoskeletal Sciences, Botnar Research Centre, Windmill Road, Oxford, OX3 7LD UK; 2 Johns Hopkins University School of Medicine, Baltimore, MD USA; 3 Nagoya City University Graduate School of Medical Sciences, Nagoya, Japan; 4 Laboratoires Lilly France, Neuilly, France; 5 Eli Lilly and Company, Indianapolis, IN USA; 6 University of Leeds School of Medicine, Leeds, UK

**Keywords:** Psoriatic arthritis, Ixekizumab, Minimal disease activity

## Abstract

**Background:**

Although psoriatic arthritis is complex and involves multiple domains, recent advances in treatments have made remission or near-remission of most symptoms a potentially achievable goal for many patients. We sought to evaluate whether achieving minimal disease activity (MDA) criteria represented meaningful improvement from the patient perspective.

**Methods:**

Data were combined from two randomized, multinational, 24 week clinical studies of ixekizumab, a high-affinity monoclonal antibody selectively targeting interleukin-17A, in biological drug-naïve or experienced adults. MDA required 5 of 7 of: tender joint count ≤1; swollen joint count ≤1; Psoriasis Area and Severity Index total score ≤ 1 or body surface area ≤ 3%; patient’s assessment of pain visual analogue scale (VAS) ≤15; patient’s global assessment of disease activity VAS ≤20; Health Assessment Questionnaire Disability Index ≤0.5; and tender entheseal points ≤ 1. MDA responders and non-responders were compared for mean change from baseline on the 36-Item Short Form Health Survey (SF-36), European Quality of Life 5 Dimension 5 Level Health Questionnaire (EQ-5D-5 L); EQ-5D-5 L VAS; and Work Productivity and Activity Impairment–Specific Health Problem (WPAI-SHP) questionnaire.

**Results:**

MDA responders had significantly greater improvements versus non-responders in each SF-36 domain and in the SF-36 physical summary score; improvements were also greater in the EQ-5D-5 L and EQ-5D-5 L VAS, and in 3 of the 4 WPAI-SHP domains. MDA responders were more likely to achieve minimal clinically important differences than non-responders.

**Conclusion:**

These findings support MDA response as being strongly associated with achieving improved disease status based on measures of patient reported health-related quality of life and productivity.

**Trial registration:**

SPIRIT-P1, NCT01695239, First Posted: September 27, 2012; and SPIRIT-P2, NCT02349295, First Posted: January 28, 2015.

**Electronic supplementary material:**

The online version of this article (10.1186/s41927-018-0030-y) contains supplementary material, which is available to authorized users.

## Background

Psoriatic arthritis (PsA) is an inflammatory musculoskeletal disease with heterogeneous clinical manifestations and significant impact on daily activities [1]. Approximately 80% of patients with PsA have psoriasis before manifesting arthritis, with the musculoskeletal disease developing on average 10 years after the onset of skin disease [2]. Clinical manifestations of PsA are frequently associated with substantial decrements in health status, including physical, emotional, and psychosocial functional disability and reduced quality of life [3, 4]. Recently, the morbidity associated with non-musculoskeletal as well as the musculoskeletal features of PsA has been better recognized [5], and led to the development of a number of composite endpoints that include evaluation of the PsA patient across the range of symptoms [6–8].

The availability of novel treatments with the potential to treat to remission or near remission has led to a paradigm shift in the therapeutic management of PsA, with treatment targets now favoring low absolute levels of disease activity across multiple disease domains rather than relative improvements [6, 9]. Of the various composite measures currently available, minimal disease activity (MDA) addresses this shift and is realistic for clinical implementation [10, 11]. MDA response has been longitudinally validated in the effect of TIght COntrol of inflammation in early Psoriatic Arthritis (TICOPA) study, a randomized treatment strategy clinical trial [12, 13]. Tight control was shown to result in more patients achieving the primary endpoint of American College of Rheumatology 20% (ACR20), and resulted in a greater median improvement in Psoriatic Arthritis Quality of Life (PsAQoL), a PsA specific quality of life measure. Achievement of MDA in PsA patients has also been shown to have prognostic value for long-term outcomes, including improved physical functioning and decrease in radiographic progression in both interventional clinical trials and observational cohorts [11, 14, 15]. It is important to understand how these improvements translate to patient-recognized benefit in order to further validate MDA as a potential composite endpoint for future PsA randomized controlled trials (RCTs).

The efficacy and safety of ixekizumab, a high-affinity monoclonal antibody selectively targeting interleukin-17A, have been evaluated in 2 phase III RCTs in patients with PsA. A significantly higher proportion of ixekizumab versus placebo patients achieved the primary endpoint at 24 weeks of ACR20, as well as numerous other efficacy endpoints, in both the biologic treatment-naïve [16, 17] and biologic experienced [18] PsA populations. The objective of the present study was to evaluate whether achievement of MDA was associated with improvements in outcomes important to PsA patients including generic health-related quality of life (HRQoL) and productivity.

## Methods

### Study design

Data were analyzed from an integrated database of 2 randomized, multinational, double-blind, placebo-controlled phase III trials investigating the efficacy and safety of ixekizumab for patients with active PsA. SPIRIT-P1 (NCT01695239) evaluated biologic disease modifying anti-rheumatoid drug (DMARD) naïve patients during a double-blind study period between January 2013 and December 2014. Patients in SPIRIT-P2 (NCT02349295) were conventional synthetic DMARD (csDMARD) experienced and previously had an inadequate response or were intolerant to tumor necrosis factor inhibitors; the double-blind study period occurred between March 2015 and September 2016. Patients were randomized to placebo (*n* = 224) or 80 mg ixekizumab every 4 weeks (IXEQ4W, *n* = 229) or every 2 weeks (IXEQ2W, *n* = 226) after a 160 mg starting dose. SPIRIT-P1 also included an adalimumab reference arm; however, data from the adalimumab arm were not analyzed in the present integrated study since there was no adalimumab arm in the SPIRIT-P2 trial.

Eligible patients were ≥ 18 years of age with a diagnosis of PsA for ≥6 months and fulfilled the Classification Criteria for Psoriatic Arthritis (CASPAR) [19]. Additional eligibility requirements for both studies included at least 3 of 68 tender joints, at least 3 of 66 swollen joints, and plaque psoriasis (current or personal history). Principal exclusion criteria were history of most types of malignant disease; recent infection requiring hospitalization or antibiotic treatment; positive test for hepatitis B, hepatitis C, or human immunodeficiency virus; or liver function or hematology test results outside of predefined limits. Primary study results and additional details on study populations and designs have been published previously [16, 18].

### Assessments

MDA was achieved if 5 of 7 criteria were met: tender joint count ≤1; swollen joint count ≤1; Psoriasis Area and Severity Index (PASI) total score ≤ 1 or body surface area ≤ 3%; patient’s assessment of pain visual analogue scale (VAS) ≤15 mm; patient’s global assessment of disease activity VAS ≤20 mm; Health Assessment Questionnaire (HAQ) Disability Index ≤0.5; and tender entheseal points ≤ 1 (assessed by the Leeds Enthesitis Index).

The primary instrument evaluated for correlation with MDA status was the 36-Item Short Form Health Survey (SF-36; higher scores indicate better functioning), with analyses focused on the 8 subscales of physical functioning, role physical, bodily pain, general health, vitality, social functioning, role emotional, and mental health. Data are also presented for the Physical Component Summary (PCS) and Mental Component Summary (MCS). Additional measures assessed include: The European Quality of Life 5 Dimension 5 Level Health Questionnaire (EQ-5D-5 L) assessing the domains of mobility, self-care, usual activities, pain/discomfort, and anxiety/depression, rating each on a scale of 1 to 5 as having “no problems, slight problems, moderate problems, severe problems, and extreme problems”); the EQ-5D VAS (0–100 scale; higher scores indicate better health); and the Work Productivity and Activity Impairment–Specific Health Problem (WPAI-SHP), assessing percentage of absenteeism, percentage of presenteeism, overall work impairment, and percentage of activity impairment outside work; higher scores indicate higher impairment). Scores for the EQ-5D-5 L were converted to a health state index score [20]; the UK algorithm was used to produce a patient-level index score between − 0.59 and 1.0 (continuous variable) using values given for the November 2014 crosswalk value sheet.

Minimum clinically important differences (MCID) were defined as a change from baseline for the SF-36 PCS and MCS scores of ≥2.5-points and for the SF-36 domain scores of ≥5 points [21, 22]; for the EQ-5D-5 L health index score of ≥0.05 points [23]; and for the EQ-5D VAS of ≥10-points [24].

### Statistical analyses

The primary study endpoint was assessed at 24 weeks; however, patients were assessed at 16 weeks for inadequate response based on fulfilling specific blinded predefined criteria for change in either tender joint count or swollen joint count from baseline. This analysis excludes inadequate responders at Week 16 and patients who discontinued before Week 24. Data for patients in the placebo and both ixekizumab arms from both RCTs comprise the integrated database used for the present analyses.

Continuous (change from baseline) analyses used a one-way ANOVA where MDA responder status is the only factor in the model. For categorical data (MCID), analyses used a chi-square if the expected value for each cell was 4 or higher; otherwise, a Fisher’s exact was used. No adjustments were made for missing data.

## Results

The overall study population included 679 patients, with 224 randomized to placebo, 226 randomized to IXEQ2W, and 229 randomized to IXEQ4W; participants who were classified as inadequate responders at Week 16 were excluded from the analysis population at Week 24 (Week 24 analysis population, *N* = 483). At baseline, mean age was 50.8 years; 53.8% of patients were female; and patients identified predominately as white (93.4%; Table 1). Mean (SD) time from PsA onset was 11.0 (9.0) years, mean CASPAR total score was 4.4 (0.9), and mean PASI total score among patients with current plaque psoriasis was 6.2 (7.5). At baseline, 59.4% of patients were csDMARD experienced and nearly half were currently receiving methotrexate (48.2%). MDA responders compared to non-responders were younger and were more likely to be male and have lower weight and body mass index (BMI).

**Table 1:** Baseline characteristics by MDA responder (−R) or non-responder (-NR) status and overall

Measure	MDA-R (*N* = 157)	MDA-NR (*N* = 326)	Total (*N* = 483)	*p*-value
Age, mean (SD)	48.2 (13.7)	52.1 (10.8)	50.8 (12.0)	0.002
Female gender, n (%)	68 (43.3)	192 (58.9)	260 (53.8)	0.001
Race, n (%)				0.121
White	143 (91.1)	307 (94.5)	450 (93.4)	
Asian	8 (5.1)	13 (4.0)	21 (4.4)	
Other	6 (3.8)	5 (1.5)	11 (2.3)	
Weight kg, mean (SD)	81.1 (17.3)	88.1 (21.2)	85.9 (20.3)	< 0.001
BMI kg/m^2^, mean (SD)	27.7 (5.1)	31.0 (7.6)	30.0 (7.0)	< 0.001
Time since PsA onset, mean years (SD)	10.5 (9.3)	11.2 (8.9)	11.0 (9.0)	0.51
Time since PsA diagnosis, mean years (SD)	7.7 (7.8)	8.5 (7.8)	8.2 (7.8)	0.63
CASPAR score, mean (SD)	4.4 (1.0)	4.4 (0.9)	4.4 (0.9)	0.96
Curr. enthesitis, n (%)	78 (50.0)	200 (61.7)	278 (57.9)	0.017
Curr. dactylitis, n (%)	47 (30.1)	63 (19.4)	110 (22.9)	0.027
Curr. psoriasis, n (%)	149 (94.9)	304 (93.3)	453 (93.8)	0.46
PASI total score^a^, mean (SD)	6.3 (6.6)	6.1 (8.0)	6.2 (7.5)	0.77
cDMARD at baseline, n (%)	93 (59.2)	194 (59.5)	287 (59.4)	0.74
Methotrexate at baseline, n (%)	77 (49.0)	156 (47.9)	233 (48.2)	0.98

^a^For patients with plaque psoriasis. *Abbreviations*, *BMI* = body mass index, *CASPAR* = classification of psoriatic arthritis; *cDMARD* = conventional disease-modifying antirheumatic drug; curr. = current, *MDA* = minimal disease activity, *NR* = non-responder, *N* = number of patients in the analysis population, *n* = number of patients in each subgroup, *PASI* = psoriasis area and severity index, *PsA* = psoriatic arthritis, −*R* = responder, *SD* = standard deviation

**Fig. 1:**
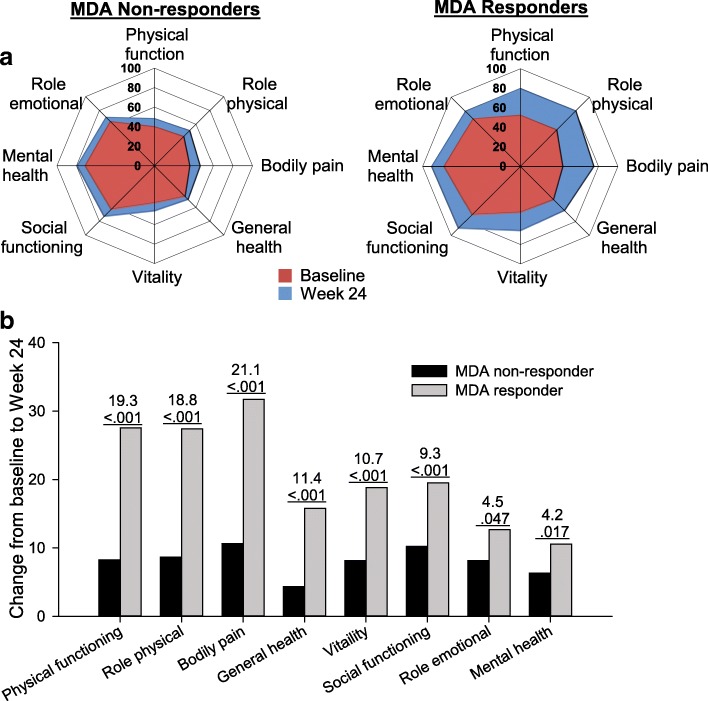
SF-36 domain scores at baseline and Week 24 and change from baseline and difference by MDA responder status. Data for the SF-36 domain scores showing (**a**) Values at Baseline (red) and Week 24 (blue) by MDA responder status and (**b**) Change from baseline to Week 24 by MDA responder status. Data labels in panel **b** show the difference and *p*-value for difference between MDA responder and non-responder groups. Note that baseline values for all SF-36 domain scores were significantly lower for MDA non-responders versus MDA responders (data shown in Additional file 1: Table S1). MDA = minimal disease activity; SF-36 = 36-Item Short Form Health Survey

At baseline, SF-36 domain scores for patients who subsequently met MDA responder criteria at 24 weeks were significantly higher than those for MDA non-responders for all domains (Fig. 1a; see Additional File 1: Table S1 for *p*-values). Following treatment, MDA responders had significantly greater improvements versus MDA non-responders in each of the 8 SF-36 domains (Fig. 1b). The magnitude of improvements for MDA responders was greatest for the physically oriented domains (bodily pain, role physical, and physical functioning; range of mean [SD] from 27.4 [25.6] to 31.7 [22.3]), followed by general health, vitality, and social functioning; range 15.8 [16.7] to 19.5 [26.9]), and was more modest, albeit still significantly greater, versus non-responders for the role emotional (12.7 [24.8]) and mental health (10.6 [20.1]) domains. In addition, the SF-36 PCS mean change from baseline was significantly higher for MDA responders versus non-responders, while the difference for the MCS was not statistically significant (Table 2).

**Table 2:** Efficacy data at baseline, Week 24, and change from baseline by MDA response, mean (SD)

	Baseline			Week 24		Change from BL			
	MDA-R (*n* = 152)	MDA-NR (*n* = 322)	*p*	MDA-R (*n* = 152)	MDA-NR (*n* = 322)	MDA-R (*n* = 152)	MDA-NR (*n* = 322)	Diff.	*p*
SF-36 PCS	35.8 (9.4)	31.9 (8.9)	< 0.001	48.1 (7.1)	35.0 (9.6)	12.3 (9.0)	3.1 (7.8)	9.2 (8.2)	< 0.001
SF-36 MCS	49.3 (11.8)	46.8 (12.6)	0.047	53.7 (8.3)	50.5 (11.3)	4.4 (11.5)	3.8 (9.7)	0.7 (10.3)	0.51
EQ-5D-5 L HSI	0.62 (.20)	0.56 (.21)	0.003	0.82 (.14)	0.63 (0.17)	0.20 (0.20)	0.07 (0.20)	0.13 (0.20)	< 0.001
EQ-5D VAS	57.5 (21.0)	53.2 (20.3)	0.040	80.0 (16.6)	58.9 (20.3)	22.5 (21.7)	5.7 (22.4)	16.8 (22.2)	< 0.001
WPAI-SHP									
% absenteeism	7.8 (20.4)^a^	6.2 (18.0)^b^	0.53	4.3 (15.1)^a^	8.2 (22.7)^b^	−3.5 (20.5)^a^	2.0 (25.7)^b^	−5.5 (23.9)	0.096
% presenteeism	33.8 (23.8)^c^	40.9 (25.6)^d^	0.061	6.8 (9.0)^c^	27.7 (22.0)^d^	−27.0 (23.3)^c^	−13.2 (27.0)^d^	−13.74 (25.67)	< 0.001
Overall work impairment	36.0 (25.7)^c^	42.2 (26.5)^d^	0.12	9.8 (13.4)^c^	30.2 (23.8)^d^	−26.2 (25.2)^c^	−12.1 (28.0)^d^	−14.1 (27.0)	< 0.001
% activity impairment non-work	42.2 (25.9)^e^	52.7 (24.9)^f^	<.001	10.0 (12.0)^e^	37.9 (24.9)^f^	−32.2 (26.9)^e^	−14.9 (28.4)^f^	−17.4 (27.9)	< 0.001

Number of patients with data available where different from total n:^a^n = 84; ^b^n = 142; ^c^n = 82; ^d^n = 134; ^e^n = 152; ^f^n = 321

*Abbreviations BL* = baseline, *Diff*. = difference, *EQ-5D-5 L* = European Quality of Life 5 Dimension 5 Level Health Questionnaire, *HSI* = Health State Index, *MCS* = mental component summary, *MDA* = minimal disease activity, *PCS* = physical component summary, *MDA-R* = MDA-responder, *MDA-NR* = MDA-non-responder, *SF-36* = 36-Item Short Form Health Survey, *VAS* = visual analog scale, *WPAI-SHP* = Work Productivity and Activity Impairment–Specific Health Problem

Additional measures that improved more for MDA responders versus non-responders included the EQ-5D-5 L Health State Index (0.20 [0.20] vs. 0.07 [0.20], respectively; *p* < .001) and the EQ-5D VAS (22.5 [21.7] vs. 5.7 [22.4]; *p* < .001). Most measures assessed on the WPAI-SHP (percentage of presenteeism, overall work impairment, and percentage of activity impairment outside of work) also showed significantly greater improvements among MDA responders versus non-responders (all *p* < .001), although the difference in percentage of absenteeism was not statistically significant (*p* = .096;

In addition to comparisons based on mean changes from baseline to 24 weeks, the percentage of patients achieving MCID on the various measures was assessed based on MDA response, as shown in Table 3. For the SF-36 domains, significantly greater odds of achieving MCID were seen for MDA responders versus non-responders for physical functioning, role physical, bodily pain, and general health; odds ratio (OR) (95% confidence interval) range 3.41 (2.14, 5.43) to 4.21 (2.55, 6.97), as well as for vitality; OR 2.60 (1.68, 4.04), and social functioning; OR 1.94 (1.30, 2.87); all *p* < .001, but not for role emotional or mental health. Participants who entered MDA status were six times more likely to achieve MCID in the SF-36 PCS score than non-responders; OR 6.21, (3.76, 10.26); *p* < 0.001, while no significant difference was seen in the SF-36 MCS score (*p* = 0.55) (Table 3). For the EQ-5D-5 L HSI and EQ-5D VAS the proportions of patients achieving MCID improvements were significantly greater for MDA responders versus non-responders (all *p* < .001).

**Table 3:** Minimal clinically important difference in change from baseline to Week 24 by MDA responder (R) or non-responder (NR) status

Achieved MCID Improvement	MDA-R (*n* = 152)	MDA-NR (*n* = 322)	*P*-Value Ɣ-Value (ASE)^a^	Odds Ratio (95% CIs)
SF-36 Domains				
Physical functioning				
Yes	124 (81.6%)	182 (56.5%)	<.001	3.41
No	28 (18.4%)	140 (43.5%)	.55 (.083)	(2.14, 5.43)
Role physical				
Yes	124 (81.6%)	175 (54.3%)	<.001	3.72
No	28 (18.4%)	147 (45.7%)	.58 (.079)	(2.34, 5.92)
Bodily pain				
Yes	130 (85.5%)	188 (58.4%)	<.001	4.21
No	22 (14.5%)	134 (41.6%)	.62 (.080)	(2.55, 6.97)
General health				
Yes	121 (79.6%)	166 (51.6%)	<.001	3.67
No	31 (20.4%)	156 (48.4%)	.57 (.077)	(2.34, 5.76)
Vitality				
Yes	118 (77.6%)	184 (57.1%)	<.001	2.60
No	34 (22.4%)	138 (42.9%)	.44 (.090)	(1.68, 4.04)
Social functioning				
Yes	95 (62.5%)	149 (46.3%)	<.001	1.94
No	57 (37.5%)	173 (53.7%)	.32 (.090)	(1.30, 2.87)
Role emotional				
Yes	66 (43.4%)	143 (44.4%)	.840	.96
No	86 (56.6%)	179 (55.6%)	−.02 (.099)	(0.65, 1.42)
Mental health				
Yes	90 (59.2%)	166 (51.6%)	.118	1.36
No	62 (40.8%)	156 (48.4%)	.15 (.097)	(0.92, 2.02)
SF-36 PCS				
Yes	130 (85.5%)	157 (48.8%)	<.001	6.21
No	22 (14.5%)	165 (51.2%)	.72 (.061)	(3.76, 10.26)
SF-36 MCS				
Yes	80 (52.6%)	160 (49.7%)	.550	1.13
No	72 (47.4%)	162 (50.3%)	.06 (.098)	(0.76, 1.66)
EQ-5D-5 L HSI				
Yes	117 (77.0%)	152 (47.2%)	<.001	3.74
No	35 (23.0%)	170 (52.8%)	.58 (.074)	(2.42, 5.78)
EQ-5D VAS				
Yes	113 (74.3%)	130 (40.4%)	<.001	4.28
No	39 (25.7%)	192 (59.6%)	.62 (.067)	(2.79, 6.56)

^a^Ɣ-values near 1 indicate a strong association between clinical response and MDA. *Abbreviations*: *ASE* = asymptotic standard error, *CIs* = confidence intervals, *EQ-5D-5 L* = European Quality of Life 5 Dimension 5 Level Health Questionnaire, *HSI* = Health State Index, *MCID* = minimal clinically important difference, *MCS* = mental component summary, *MDA* = minimal disease activity, *PCS* = physical component summary, *SF-36* = 36-Item Short Form Health Survey, *VAS* = visual analog scale

## Discussion

As PsA treatment paradigms evolve, clinical trials are increasingly measuring multiple disease manifestations and aspects of life impact. Although therapeutic options are increasing, a significant number of PsA patients do not achieve uniform improvements indicating an unmet need for outcome prediction strategies to inform our current approach to treatment. MDA has been identified as an optimal state to aim for in treating PsA [25].

In the present study, we hypothesized that based on reduced pain and physical symptoms, MDA responders would experience greater improvements than non-responders in physical, emotional, and psychosocial outcomes, including domains of importance to patients such as the ability to work and impairment at home [26]. Indeed, MDA responders demonstrated significantly greater improvements than non-responders in all SF-36 domains, with the greatest improvements in physical functioning, role physical, and bodily pain. A significant improvement was also seen for the SF-36 PCS, but not MCS, as discussed below. Significantly greater improvements were seen for MDA responders versus non-responders in the EQ-5D-5 L Health State Index and EQ-5D VAS, supporting the value of MDA response as a predictor of health utility. MDA responders also had significantly greater improvements versus non-responders in presence at work, overall work impairment, and percentage of activity impairment outside of work, as assessed by the WPAI-SHP instrument. Proportions of patients achieving MCID cutoffs were significantly greater in MDA responders versus non-responders, with differences seen for 6 of the 8 SF-36 domains (all domains except role emotional and mental health), the SF-36 PCS, and for the EQ-5D-5 L Health State Index and EQ-5D VAS.

A significant improvement was not seen for the SF-36 MCS in this study. Notably, the SF-36 summary scores are calculated as a weighted sum of all 8 domains, but for the MCS, the mental domains are weighted positively and the physical domains negatively; given that the physical domains showed the greatest improvement with MDA response, this may have prevented the detection of an effect on the MCS. Indeed on further analysis, at Week 24 all eight component scores showed significantly greater improvement from baseline among MDA responders versus nonresponders (data not shown), suggesting that the negative weighting in MCS may be the issue. Such inconsistencies have led to the recommendation that conclusions should not be drawn solely on PCS and MCS scores without taking individual domain scores into consideration [27].

Some differences were noted between the populations of patients who became responders and those who did not. At baseline, patients who became MDA responders had better HRQoL, were younger, more likely to be male, and had lower body mass index (BMI) compared to non-responders. This is consistent with observations from longitudinal studies of tumor necrosis factor inhibitors that higher BMI is associated with a lower treatment response [28]. Although MDA non-responders had significantly lower HRQoL scores across measures at baseline, their magnitude of improvement and percentage achieving MCID were lower than for MDA responders. This indicates a need to better characterize this subset of PsA patients who appear to have worse disease impact and to be less responsive to treatment. Finally, although enthesitis at baseline was associated with subsequent MDA nonresponse, while baseline dactylitis was associated with subsequent MDA response, it is likely that these findings reflect a relationship with the specific measures defining MDA (which include enthesitis count and tender/swollen joint count) rather than prediction of disease activity.

The results from these ixekizumab RCTs are supported by recent findings from small cross-sectional studies. Patients not in MDA from the Netherlands scored significantly worse on measures of symptoms and functioning (BASDAI, HAQ), dermatology quality of life index (DLQI), daily activity impairment (WPAI ADL), and the mental and physical components of the SF-36 [29]. Queiro and colleagues assessed the association between MDA status and PsA life impact as assessed by the PsA Impact of Disease (PsAID) questionnaire among Spanish patients fulfilling CASPAR criteria with disease for at least 1 year [30]. Among the 58.6% of patients with MDA, the impact of disease was significantly lower based on mean PsAID score (instrument range 0 to 10; MDA responders 3.3 vs. non-responders 7.1; *p* < .0001) and also based on the proportion of patients in the PsAID patient acceptable symptom state of < 4 [31] (66.7% of MDA responders vs. 37.4% of non-responders; *p* < .0001).

A major strength of our study, in addition to MDA being pre-specified as a key secondary outcome in one of the studies, was the availability of pre- and post-treatment data, which enabled us to compare MDA responders versus non-responders in terms of the magnitude of change during treatment, as well as the percentages of individuals who achieved MCID in improvements from baseline. A limitation of our study is the exclusion of patients who were inadequate responders at Week 16 due to the study design, which specified a potential change in therapy at Week 16 for patients not meeting pre-defined minimal response criteria, while MDA was assessed at baseline and at 24 weeks. However, inadequate responders would presumably include more MDA non-responders than responders and their exclusion is most likely to lessen the differences between groups. Another limitation is that the analyses were performed post hoc.

## Conclusions

In this study assessing both biologic naïve and experienced PsA patients treated with ixekizumab in two phase III RCTs, MDA response was found to be associated with significantly greater improvements in all SF-36 domains, the SF-36 PCS but not MCS score, and both the EQ-5D-5 L Health State Index and VAS scores. In addition, greater improvements in work-related functioning on the WPAI were seen in MDA responders versus non-responders. These results indicate that MDA response was strongly associated with improvements in generic HRQoL and productivity in patients with PsA. Taken together with previous findings showing improved physical functioning and reduced disease progression on MDA responders, these findings suggest that MDA response is a strong discriminator for achieving a desirable disease status based on both physician and patient reported outcomes in PsA.

## Additional file

Additional file 1: **Table S1.** SF-36 domain scores by MDA responder status, mean (SD) and *p*-values. (DOCX 15 kb)

## Abbreviations

ACR20: American College of Rheumatology 20%
ANOVA: Analysis of variance
BASDAI: Bath Ankylosing Spondylitis Disease Activity Index
BMI: Body mass index
CASPAR: Classification Criteria for Psoriatic Arthritis
CI: Confidence interval
csDMARD: Conventional synthetic DMARD
DLQI: Dermatology quality of life index
DMARD: Disease modifying anti-rheumatoid drug
EQ-5D-5 L: European Quality of Life 5 Dimension 5 Level Health Questionnaire
HAQ: Health Assessment Questionnaire
HRQoL: Health-related quality of life
IxeQ2W: 80 mg IXE ixekizumab every 2 weeks
IxeQ4W: 80 mg IXE ixekizumab every 4 weeks
MCID: Minimum clinically important differences
MCS: Mental Component Summary
MDA: Minimal disease activity
OR: Odds ratio
PASI: Psoriasis Area and Severity Index
PCS: Physical Component Summary
PsA: Psoriatic arthritis
PsAID: PsA Impact of Disease
PsAQoL: Psoriatic Arthritis Quality of Life
RCTs: Randomized controlled trials
SF-36: Short Form Health Survey
TICOPA: TIght COntrol of inflammation in early Psoriatic Arthritis
VAS: Visual analogue scale
WPAI ADL: WPAI daily activity impairment
WPAI-SHP: Work Productivity and Activity Impairment–Specific Health Problem
