# Supplementary material for: Achieving minimal disease activity in psoriatic arthritis predicts meaningful improvements in patients’ health-related quality of life and productivity
Source: BMC Rheumatol. 2018 Aug 13;2:24. doi: 10.1186/s41927-018-0030-y (PMC6390571; doi:10.1186/s41927-018-0030-y)
Supplement: Supplementary file 1 — Table S1. SF-36 domain scores by MDA responder status, mean (SD) and p-values. (DOCX 15 kb) [file 41927_2018_30_MOESM1_ESM.docx]

**Additional file 1 Table S1 SF-36 domain scores by MDA responder status, mean (SD) and p-values**

|  | **Baseline** | | | | **Week 24** | | **Change from Baseline** | | | |
| --- | --- | --- | --- | --- | --- | --- | --- | --- | --- | --- |
|  | **MDA-R**  **(*n*=152)** | **MDA-NR**  **(*n*=322)** | **Total** | **p-value** | **MDA-R**  **(*n*=152)** | **MDA-NR**  **(*n*=322)** | **MDA-R**  **(*n*=152)** | **MDA-NR**  **(*n*=322)** | **Difference** | **p-value** |
| Physical functioning | 52.8 (25.8) | 40.6 (25.0) | 44.5 (25.9) | <0.001 | 80.3 (17.6) | 48.9 (25.1) | 27.6 (24.3) | 8.2 (19.1) | 19.3 (20.9) | <0.001 |
| Role physical | 53.3 (26.8) | 42.9 (24.3) | 46.2 (25.6) | <0.001 | 80.8 (21.3) | 51.5 (25.9) | 27.4 (25.6) | 8.7 (22.2) | 18.8 (23.3) | <0.001 |
| Bodily pain | 44.1 (20.6) | 36.6 (18.2) | 39.0 (19.3) | <0.001 | 75.8 (16.0) | 47.2 (19.7) | 31.7 (22.3) | 10.6 (20.3) | 21.1 (21.0) | <0.001 |
| General health | 48.5 (20.6) | 44.7 (18.7) | 45.9 (19.4) | 0.049 | 64.3 (20.7) | 49.1 (20.2) | 15.8 (16.7) | 4.3 (16.1) | 11.4 (16.3) | <0.001 |
| Vitality | 47.5 (20.7) | 38.5 (20.9) | 41.4 (21.2) | <0.001 | 66.4 (19.6) | 46.7 (21.4) | 18.8 (23.6) | 8.2 (18.7) | 10.7 (20.4) | <0.001 |
| Social functioning | 70.7 (25.9) | 63.6 (26.9) | 65.9 (26.8) | 0.008 | 90.2 (16.7) | 73.8 (24.8) | 19.5 (26.9) | 10.2 (23.9) | 9.3 (24.9) | <0.001 |
| Role emotional | 78.8 (25.2) | 71.9 (26.9) | 74.1 (26.6) | 0.011 | 91.5 (16.5) | 80.1 (23.7) | 12.7 (24.8) | 8.2 (22.2) | 4.5 (23.0) | 0.047 |
| Mental health | 69.4 (21.3) | 64.8 (21.2) | 66.3 (21.3) | 0.020 | 80.0 (16.2) | 71.1 (19.0) | 10.6 (20.1) | 6.3 (16.9) | 4.2 (18.0) | 0.017 |

Abbreviations: MDA= minimal disease activity; MDA-R= MDA responder; MDA-NR= MDA non-responder; SF-36 = 36-Item Short Form Health Survey.
